# Maximum-entropy and subspace methods for high-resolution relaxation-diffusion distribution estimation

**DOI:** 10.1162/IMAG.a.113

**Published:** 2025-08-19

**Authors:** Lipeng Ning

**Affiliations:** Brigham and Women’s Hospital, Boston, MA, United States; Harvard Medical School, Boston, MA, United States

**Keywords:** quantitative relaxometry, diffusion MRI, diffusion-relaxation distribution, maximum entropy, subspace

## Abstract

Relaxation-diffusion distribution characterizes tissue microstructure using multi-contrast MRI data without using a multi-compartment model. This work applies and generalizes two nonlinear spectral estimation algorithms to compute relaxation-diffusion distributions and compares their performances with the standard linear inverse method. The first algorithm employs maximum entropy (MaxEnt) estimation, extending previous methods by incorporating measurement noise for improved robustness. The second algorithm is based on the MUltiple SIgnal Classification (MUSIC) subspace spectral estimation technique, enabling pseudo-spectral estimation of multi-exponential signals sampled on regular grids without solving optimization problems. Both methods were compared against the basis representation technique and the nonnegative least squares (NNLS) method using synthetic and *in vivo* data. MaxEnt demonstrated superior spectral resolution compared to other methods. Meanwhile, the multidimensional MUSIC algorithm provided accurate estimations but required a higher signal-to-noise ratio. MaxEnt and MUSIC improve computational efficiency, especially when a high-resolution sampling grid is required for the density functions.

## 1 Introduction

Magnetic resonance imaging (MRI), such as $T_{1}$-weighted ($T_{1w}$ ) and $T_{2}$-weighted ($T_{2w}$ ) MRI, provides different contrasts between tissue types to investigate the tissue structure and diagnose diseases (Lerch et al., 2017; Mugler III & Brookeman, 1990). Quantitative relaxometry improves $T_{1w}$ and $T_{2w}$ MRI to measure the $T_{1}$ and $T_{2}$ coefficients using data acquired with multiple sequence parameters (Cheng et al., 2012; Deoni, 2010; Deoni et al., 2005; Fujita et al., 2019; Marques et al., 2010). Diffusion MRI (dMRI) provides complementary information to quantitative relaxometry about the microstructure of the cellular arrangement in the underlying tissue (Basser et al., 1994; Stejskal & Tanner, 1965). While diffusion MRI and relaxometry images are usually acquired and analyzed separately in neuroimaging research (Does & Snyder, 1996; Does et al., 1998; Kaden et al., 2016; Ning et al., 2015, 2017; Palombo et al., 2020; H. Zhang et al., 2012), joint analysis of multi-contrast MRI data in a high-dimensional space of relaxation and diffusion can provide more specific information about tissue microstructure such as component-specific $T_{1}$ and $T_{2}$ relaxometry parameters (Chan & Marques, 2020; Chatterjee et al., 2018; Deoni et al., 2008; Lancaster et al., 2003; Raj et al., 2014), the relaxation and diffusion parameters (Gong et al., 2020; Veraart et al., 2018), or the correlation coefficients between relaxation and diffusion (Ning et al., 2020). Multi-compartment models are usually developed to estimate compartment-specific tissue parameters. However, the models depend on a priori assumptions of signal models, the number of compartments, and constraints on model parameters. Moreover, the estimation algorithm usually has the degeneracy issue related to locally optimal solutions (Jelescu et al., 2016; West et al., 2019).

An alternative approach for microstructure analysis is to estimate the continuous distribution of relaxometry coefficients (Kroeker & Mark Henkelman, 1986; Whittall & MacKay, 1989; Wiggermann et al., 2020; Yu et al., 2021) or the continuous distribution of diffusion coefficients (Periquito et al., 2021; Pfeuffer et al., 1999). This approach is generalized to the diffusion-relaxation correlation spectroscopy method to estimate the joint distribution of multiple tissue parameters in a high-dimensional space, such as the $T_{1}$-$T_{2}$, $T_{1}$-Diffusion, $T_{2}$-Diffusion, and $T_{1}$-$T_{2}$-Diffusion correlation spectroscopy methods (Avram et al., 2021; Benjamini et al., 2021, 2023; Endt et al., 2023; Kim et al., 2017, 2020; Martin et al., 2021; Ning et al., 2020; Slator et al., 2021; Topgaard, 2017). For simplicity, these methods will be collectively referred to as a relaxation-diffusion distribution (RDD) in this paper, which may represent distributions in any one or multidimensional subspace. Continuous relaxation-diffusion distributions provide a non-parametric approach to determining the number of tissue compartments and their tissue property. These methods are initially developed to investigate the microstructure of porous medium (Bernin & Topgaard, 2013; Callaghan et al., 2003; de Almeida Martins & Topgaard, 2018; Hürlimann et al., 2002; Montrazi et al., 2020; Song et al., 2002). Recent fast MRI acquisition techniques (Avram et al., 2021; Hutter et al., 2018; Ji et al., 2021, 2022) significantly reduce the scan time to gradually make relaxation-diffusion distribution methods feasible for *in vivo* human scans and neuroimaging research. The goal of this paper is to apply and generalize two nonlinear methods for RDD estimation and compare their performance with the standard linear inverse model.

Basis representation is a standard approach for estimating RDD using finite MRI measurements, where the basis matrix contains signals computed on a discrete set of parameters. The measured MRI data vector is represented by the basis matrix using a vector of representation coefficients. The number of basis functions is typically much larger than the number of measurements. Thus, the representation vector is estimated by solving an ill-posed inverse problem with a suitable algorithm such as the nonnegative least squares (NNLS) method (Benjamini & Basser, 2016; Kim et al., 2017; Wiggermann et al., 2020). This approach has several well-known limitations. The basis matrix depends on a pre-selected discrete set of tissue parameters. A low-resolution basis matrix can lead to inaccurate representations of off-the-grid spectral content (Chi et al., 2011), reducing the spectral resolution and the accuracy of the estimated tissue parameters. Increasing the number of elements in the basis matrix is usually not an efficient approach to boost the spectral resolution since it will worsen the coherence between the basis functions and escalate the computation complexity to solve the inverse problem, making it impossible to solve the NNLS problem within any reasonable time. Monte Carlo inversion methods have been proposed to improve the computation efficiency by drawing random samples in the parameter spaces (de Almeida Martins & Topgaard, 2018; Martin et al., 2021; Narvaez et al., 2022; Prange & Song, 2009; Topgaard, 2019). For large-dimensional spaces, these methods typically require a trade-off between the number of samples and the randomness of the results. Many previous studies have used low-resolution dictionary matrices with an ad-hoc selection of spectral resolution for correlation spectroscopy analysis with exact optimal solutions. For example, the method in Avram et al. (2021) uses 144 basis functions based on 12 samples in D and 12 in $T_{1}$ to estimate the $T_{1}$-D correlation spectroscopy. In Endt et al. (2023), three dictionaries with 3600 basis functions were used separately to compute the $T_{1}$-$T_{2}$, $T_{1}$-D, and $T_{2}$-D distributions (60 samples in each dimension) without a joint three-dimensional representation of the density functions. A more efficient approach is needed for estimating high-resolution RDD analysis.

This work applies and generalizes two new methods for RDD estimation based on techniques from nonlinear spectral estimation. The first method is based on the maximum-entropy (MaxEnt) estimation approach, a classical technique for solving inverse problems in probability theory and power spectral estimation (Cover & Thomas, 2006; Jaynes, 1982). A well-known result in signal processing is that the MaxEnt power spectrum of a stationary time series with a finite set of autocovariances is represented by an auto-regressive model (Burg, 1975; Haykin, 1983). The Gaussian probability distribution is the MaxEnt distribution with fixed mean and variance in probability theory (Cover & Thomas, 2006). MaxEnt methods have been applied for the estimation of nuclear magnetic resonance spectroscopy (Heaton, 2005; Hoch, 1985; Rathi et al., 2014; Sibisi et al., 1984) and $T_{1}$-$T_{2}$ distribution (Chouzenoux et al., 2010). The method in Chouzenoux et al. (2010) relies on solving a large-scale convex optimization problem to directly estimate the density function, which is inefficient for large-dimensional data. The method in Mitchell et al. (2012), which is based on Heaton (2005), estimates the optimal parameters of the maximum-entropy distribution in a much lower-dimensional space to improve computational efficiency. However, the algorithm requires solving a non-convex nonlinear least squares problem. Our previous work (Ning, 2023) introduces a dual formulation for MaxEnt RDD estimation, which involves a convex optimization problem in a low-dimensional space. In this work, we further extend the method in Ning (2023) and derive a dual formulation for a problem similar to Chouzenoux et al. (2010) to integrate measurement noise to improve the robustness of the estimation MaxEnt distribution by solving efficient convex optimization problems.

The second approach uses a generalized Multiple Signal Classification (MUSIC) spectral estimation method to characterize multi-exponential MRI signals. MUSIC and MaxEnt both belong to the family of nonlinear power spectral estimation algorithms to improve the spectral resolution of Fourier transform-based methods (Haykin, 1983; Stoica et al., 1997). The MUSIC method has been generalized to multidimensional spectral estimation in Hua (1993); Liao (2015) and has been applied for NMR estimation (Li et al., 1998). This work introduces a modified multidimensional MUSIC method for MRI signals of $T_{1}w$ contrast with varying inversion recovery times. The MUSIC method can localize the peak locations of the RDD functions without solving optimization problems, a major advantage compared to other methods. However, it requires samples on a regular grid, which may significantly increase the scan time in practice.

The following sections will introduce more details on the background, the MaxEnt method, the MUSIC method, experiments, and comparison results for different simulation *in vivo* data methods. Small letters, such as x and θ, denote scalars for notations. Boldface letters, such as x,θ , denote vectors. Capital letters are used to either denote matrices, such as A, B, or integer numbers, such as K and N, whose meanings are self-contained within the context.

## 2 Theory

### 2.1 Problem formulation

For a general signal model, let $\theta \;=\;[\theta_{1};\theta_{2},...,\theta_{d}]\in {ℛ}^{d}$ denote a d-dimensional vector that represents tissue microstructure parameters, such as a vector that consists of the $T_{1}$, $T_{2}$, $T_{2}^{*}$ relaxation time, or diffusion coefficient D. Let $x\in {ℛ}^{d}$ denote the measurement parameters, such as the echo time (TE), repetition time (TR), inversion recovery time (TI), or the b-values. Let a(x;θ) denote the signal contribution from a tissue compartment characterized by θ. Then, the measured signal is represented as

$$s(\text{x})=\int_{\Theta }a(\text{x};\theta )\rho (\theta )d\theta +\epsilon ,$$ (1)

where ρ denotes a density function over a compact parameter space Θ and ϵ denotes the measurement noise. If ρ consists of a sum of multiple Dirac delta functions, such as

$$\rho (\theta )=\sum_{k=1}^{K}c_{k}\delta (\theta -\theta_{k}),$$ (2)

then Eq. (1) becomes the so-called multi-compartment models.

The density function ρ provides a non-parametric characterization of the tissue microstructure. The problem considered in this paper is to estimate the density ρ given a finite set of measures $s(x_{i})$ for i=1,...,N with known functions a(x;θ). Suppose the signal source is from multiple tissue types. In that case, the number of dominant peaks in the density function estimates the number of compartments, with the tissue parameters given by the peak locations.

The general formulation in Eq. (1) has been widely used in many applications to estimate the RDD functions where the distribution is calculated using the basis representation techniques. In these methods, the N dimensional signal vector s is represented as s=Ax with $A\in {ℛ}^{N\times M}$ where each column of the basis matrix A represents signals from a single compartment. The vector x can be estimated by solving the inverse problem, using, for example, the nonlinear least squares (NNLS) algorithm, to provide a discrete representation of the density functions. The choice of the basis matrix is critical for the estimation results. High-resolution results require significantly higher computational complexity, especially for multidimensional data analysis. For example, assume that K samples are selected in each parameter space dimension Θ, then a total number of $M=K^{d}$ basis functions are needed. The increased size of the basis matrix significantly raises the computational complexity in solving the inverse problem to estimate x. The following sections introduce two alternative computationally efficient methods to solve the inverse problem in Eq. (1).

### 2.2 Maximum-entropy estimation

#### 2.2.1 The dual formulation

The problem of estimating ρ(θ) given a finite number of measurements of $s(x_{n})$ is closely related to the moment problem in probability theory and spectral analysis. Maximum entropy (MaxEnt) estimation is a classical technique for solving the moment problem (Ambrozie, 2004; Burg, 1975; Mead & Papanicolaou, 1984) and has been applied in several applications in MRI and NMR. A general formulation for estimating ρ(θ) for finite measurements is given below:

$$\begin{array}{cccc} & \underset{\rho \left(\theta \right)}{\text{max}}-\int_{\Theta }\rho (\theta )\;\text{ln }\rho (\theta )d\theta & & \\ \text{s.t.} & \int_{\Theta }a_{n}(\theta )\rho (\theta )d\theta =s_{n},\;\text{for }n=1,...,N, & & \end{array}$$ (3)

where $a_{n}(\theta )=a(x_{n};\theta )$ for simplicity, and the objective function quantifies the Shannon differential entropy of ρ(θ) .

The MaxEnt approach has led to several well-known analytic expressions, including the Normal distribution in moment problems with given mean and variances (Cover & Thomas, 2006), and the autoregressive model is the MaxEnt solution for a stochastic process with constraints on a finite autocorrelation series (Burg, 1975; Haykin, 1983).

The MaxEnt method has been applied to estimate $T_{1}$-$T_{2}$ distribution in Chouzenoux et al. (2010) by solving an entropy-regularized inverse problem in the high-dimensional space of discrete density functions. Our previous work in Ning (2023) introduced a dual formulation for the MaxEnt problem, which led to an analytic expression to reduce the dimension of the optimization variables and improve the computation complexity. The dual problem introduced in Ning (2023) has the following expression

$$\begin{array}{ccc} \underset{\lambda_{n}}{\text{min}}\int \text{exp}\left(-\;\sum_{n=1}^{N}\lambda_{n}a_{n}(\theta )\right)d\theta +\;\sum_{n=1}^{N}\lambda_{n}s_{n}, & & \end{array}$$ (4)

where $\lambda_{n}$ for n = 1,...,N are the dual variables. For simplicity, let $\lambda =\;[\lambda_{1};\lambda_{2};..\;.;\lambda_{N}]$ , $a(\theta )\;=\;[a_{1}(\theta );a_{2}(\theta );...;a_{N}(\theta )]$ , $s=\;[s_{1};s_{2},...,s_{N}]$ . Then, (4) is simplified as

$$\begin{array}{ccc} \underset{\lambda }{\text{min}}\int \text{exp}\left(-\;\lambda_{n}\cdot a\left(\theta \right)\right)d\theta +\lambda \cdot s, & & \end{array}$$ (5)

where ⋅ denotes the inner product.

Let $\hat{\lambda }$ denote the optimal solution; then, the MaxEnt density function is given by Ning (2023)

$$\begin{array}{ccc} \rho_{\text{MaxEnt}}(\theta )=\text{exp}(-\hat{\lambda }\cdot a(\theta )-1). & & \end{array}$$ (6)

The above dual formulation was applied to estimate $T_{2}$-Diffusion distribution. The same approach can be applied to RDD analysis of arbitrary MRI signal models. However, a limitation of the formulation in Eq. (3) is that the measurement noise is ignored, which is not satisfied in practice, making the results sensitive to noise. A general formulation is introduced below to incorporate measurement noise in the MaxEnt problem.

#### 2.2.2 MaxEnt estimation with noisy data

To further extend Eq. (3) by incorporating measurement noise, a generalized formulation is introduced as follows

$$\begin{array}{cccc} & \underset{\rho (\theta ),\hat{s}}{\text{max}}-\int_{\Theta }\rho \left(\theta \right)\;\text{ln }\rho \left(\theta \right)d\theta -\frac{1}{2\sigma }∥\hat{s}-s{∥}_{2}^{2} & & \\ & \text{s.t.}\int_{\Theta }a_{n}\left(\theta \right)\rho \left(\theta \right)d\theta ={\hat{s}}_{n},\;\text{for }n=1,...,N, & & \end{array}$$ (7)

where the auxiliary variable $\hat{s}\;=\;[{\hat{s}}_{1};...;{\hat{s}}_{N}]$ is introduced to mitigate the discrepancy between the density function and measurements because of noise. The nonnegative parameter σ is selected based on the level of measurement noise. The maximization of the negative quadratic term in Eq. (7) may seem uncommon, but this formulation simplifies the derivation of the dual formulation below. In probability theory, the entropy of a probability distribution can be interpreted as the log-likelihood over the space of all random probability distributions that satisfy the constraints (Jaynes & Bretthorst, 2003; Van Campenhout & Cover, 1981). In this framework, Eq. (7) can be interpreted as the maximum log-likelihood of the joint probability of the density function and measurement noise, assuming the noise is i.i.d. Gaussian noise, considering the density function has a unit integral. This interpretation suggests a heuristic choice for σ as $\sigma \approx \text{var(}\epsilon \text{)}/\int_{\Theta }p\left(\theta \right)d\theta$ . For a sufficiently small positive σ, the optimal ${\hat{s}}_{n}$ is expected to be close to $s_{n}$, similar to the noiseless case in the above subsection.

The Lagrangian of Eq. (7) is given by Eq. (7):

$$\begin{array}{ccc} \begin{array}{l} L\left(\rho ,\hat{s},\lambda \right)=-\int_{\Theta }\rho \left(\theta \right)\;\text{ln }\rho \left(\theta \right)d\theta -\frac{1}{2\sigma }∥\hat{s}-s{∥}_{2}^{2}\\ \;\;\;\;\;\;\;\;\;\;\;\;\;\;\;\;\;\;\;\;\;\;\;\;\;\;\;\;+\;\sum_{n=1}^{N}\lambda_{n}\left(\int_{\Theta }a_{n}\left(\theta \right)\rho \left(\theta \right)d\theta -{\hat{s}}_{n}\right), \end{array} & & \end{array}$$ (8)

where λ denotes the multipliers. Then, the optimization problem Eq. (7) is obtained as the solution to the following problem

$$\begin{array}{ccc} \underset{\rho ,\hat{s}}{\text{max}}\underset{\lambda }{\text{min}}L\left(\rho ,\hat{s},\lambda \right)=\underset{\lambda }{\text{min}}\underset{\rho ,\hat{s}}{\text{max}}L\left(\rho ,\hat{s},\lambda \right). & & \end{array}$$ (9)

Based on variational analysis of L, the optimal ρ and $\hat{s}$ should satisfy that

λ⋅a(θ)−ln ρ(θ)−1=0, (10)

$$\frac{1}{\sigma }(\hat{s}-s)+\;\lambda =0.$$ (11)

Thus, the optimal ρ and $\hat{s}$ for a given set of λ are equal to

ρ(θ;λ)=exp(λ⋅a(θ)−1), (12)

$$\hat{s}=s-\sigma \lambda .$$ (13)

By substituting Eqs. (12) and (13) to the right hand side of Eq. (9), the dual optimization problem is obtained below:

$$\begin{array}{c} \underset{\lambda }{\text{min}}\int_{\Theta }\text{exp}\left(\lambda \cdot a\left(\theta \right)-1\right)\;d\theta -\lambda \cdot s+\frac{\sigma }{2}∥\lambda {∥}_{2}^{2}. \end{array}$$ (14)

The objective function in Eq. (14) becomes the same as in Eq. (4) if σ = 0 in the noiseless case. Let $\hat{\lambda }$ denote the optimal solutions to Eq. (14), then the optimal ρ(θ) has the same analytic expression given by Eq. (6).

For completeness, the gradient and Hessian of the objective function of Eq. (14) are derived below, which can be used to derive optimization algorithms and prove the convexity of Eq. (14). Let

$$\begin{array}{c} g\left(\lambda \right)=\int_{\Theta }\text{exp}\left(\lambda \cdot \text{a}\left(\theta \right)-1\right)\;d\theta -\lambda \cdot s+\frac{\sigma }{2}∥\lambda {∥}_{2}^{2}, \end{array}$$ (15)

denote the objective function. Then, the first- and second-order derivative of g(λ) are given by

$$\frac{\partial g\left(\lambda \right)}{\partial \lambda_{i}}=\int_{\Theta }a_{i}\left(\theta \right)\rho \left(\theta ;\lambda \right)d\theta -s_{i}+\sigma \lambda_{i},$$ (16)

$$\frac{\partial^{2}g\left(\lambda \right)}{\partial \lambda_{i}\partial \lambda_{j}}=\int_{\Theta }a_{i}\left(\theta \right)a_{j}\left(\theta \right)\rho \left(\theta ;\lambda \right)d\theta +\sigma \delta_{ij},$$ (17)

where $\delta_{ij}$ denotes the Kronecker delta function. Eq. (17) shows that the function g(λ) is strictly convex. In practice, the derivatives require a numerical implementation of the integrals. The optimization problem can be solved efficiently using the Newton algorithm, which only involves the inversion of an N×N Hessian matrix, which is an advantage compared to the solution for basis-representation techniques that usually require computing the inversion of a large-scale matrix.

There are several theoretical advantages to introducing the auxiliary variable and measurement noise in Eqs. (7) and (14) compared to the original formulations in Eqs. (3) and (4). First, the auxiliary variables ensure that the primal optimization problem is strictly feasible, whereas the original primal problem Eq. (3) may not have a feasible solution if measurement noise exists. The strict feasibility of the constraint in Eq. (7) ensures that there is no duality gap between the primal and dual problems, so that the dual problem has a bounded optimal value. Second, the additional quadratic term introduces an identity matrix $\sigma I_{N\times N}$ in the Hessian matrix to improve the condition number so that the inversion of the Hessian matrix is well-posed, which may not hold for the noiseless formulation in Eq. (4).

### 2.3 MUSIC subspace method

This section introduces an alternative approach for correlation spectroscopic analysis of multi-exponential MRI signals. Exponential functions are often used for modeling signals in several MRI modalities, such as multi-echo MRI, diffusion MRI signal, and the combined $T_{2}$-relaxation and diffusion MRI techniques (Liang & Lauterbur, 2000; Mori & Tournier, 2014). In power-spectral analysis, the subspace methods consist of a family of approaches, including the MUltiple SIgnal Classification (MUSIC) method, that use the singular-value decomposition to decompose the measurements into the signal and noise subspace to estimate the pseudo spectra for power spectral analysis for trigonometric or exponential signals. The MUSIC subspace method does not require the solution of an optimization problem or the selection of a regularization parameter. In the following subsection, we introduce the background of the MUSIC method, a tailored MUSIC method for a special type of signal model for $T_{1}$-weighted MRI with varying TI times, and a generalized MUSIC method for multidimensional RDD analysis.

#### 2.3.1 The MUSIC method

Consider the following noiseless univariate multi-exponential signal model

$$\begin{array}{ccc} {\hat{s}}_{n}=\;\sum_{k=1}^{K}c_{k}e^{-\theta_{k}x_{n}},\;\text{for }n=1,...,N, & & \end{array}$$ (18)

where the measurement parameters $x_{1},...,x_{N}$ are sampled on a regular grid, for example, $x_{n+1}-x_{n}\;=\;\delta_{x}$. For a sufficiently large number of measurements, that is, N>2 K , the following Hankel matrix

$$H\left(\hat{s}\right)=\left[\begin{array}{cccc} {\hat{s}}_{1} & {\hat{s}}_{2} & ... & {\hat{s}}_{K+1}\\ {\hat{s}}_{2} & {\hat{s}}_{3} & ... & {\hat{s}}_{K+2}\\ ⋰ & ⋰ & ⋰ & \vdots \\ {\hat{s}}_{N-K} & {\hat{s}}_{N-K+1} & ⋰ & {\hat{s}}_{N} \end{array}\right]$$ (19)

$$=\left[\begin{array}{ccc} e^{-\theta_{1}x_{1}} & \cdots & e^{-\theta_{K}x_{1}}\\ e^{-\theta_{1}x_{2}} & \cdots & e^{-\theta_{K}x_{2}}\\ \vdots & \vdots & \vdots \\ e^{-\theta_{1}x_{N-K}} & \cdots & e^{-\theta_{K}x_{N-k}} \end{array}\right]\left[\begin{array}{cccc} c_{1} & c_{1}e^{-\theta_{1}\delta_{x}} & \cdots & c_{1}e^{-K\theta_{1}\delta_{x}}\\ c_{2} & c_{2}e^{-\theta_{2}\delta_{x}} & \cdots & c_{2}e^{-K\theta_{2}\delta_{x}}\\ \vdots & \vdots & \vdots & \vdots \\ c_{K} & c_{K}e^{-\theta_{K}\delta_{x}} & \cdots & c_{2}e^{-K\theta_{K}\delta_{x}} \end{array}\right]$$ (20)

which is singular with $\text{rank}\;(\text{H}(\hat{s}))=\;\text{K}$ and the two matrix factors are non-singular as long as $\theta_{i}\neq \theta_{j}$ if i≠j . Define the following signal vector

$$\begin{array}{c} e\left(\theta \right)=[e^{-\theta x_{1}};e^{-\theta x_{2}};...;e^{-\theta x_{N-K}}]. \end{array}$$ (21)

Then, $e(\theta )\in \text{span}(H(\hat{s}))$ for $\theta \;=\;\theta_{1},...,\theta_{K}$.

If measurement noise exists, that is, $s_{n}\;=\;{\hat{s}}_{n}+w_{n}$, then the Hankel matrix H(s) is not singular. The MUSIC approach is developed based on the assumption that the signal vector $e(\theta_{k})$ is closer to the signal subspace than the noise subspace. Specifically, let U(H(s)) denote an orthogonal basis of the column space based on the singular value decomposition, which is decomposed as

$$\begin{array}{c} U(H(s))=[U_{s},\;U_{n}] \end{array}$$ (22)

with $U_{s}$ corresponding to the K largest singular values. Thus, $U_{s}$ and $U_{n}$ are orthogonal bases for the signal and the noise subspaces. The MUSIC (pseudo) density function is defined as

$$\begin{array}{ccc} \rho_{\text{MUSIC}}\left(\theta \right)=\frac{e{\left(\theta \right)}^{T}e\left(\theta \right)}{e{\left(\theta \right)}^{T}U_{n}U_{n}^{T}e\left(\theta \right)}, & & \end{array}$$ (23)

where the numerator is introduced to normalize the testing signal vector e(θ) . Since the signal vector Eq. (21) is orthogonal to the noise in the ideal case, the function $\rho_{\text{MUSIC}}(\theta )$ is expected to have peaks at $\theta_{k}$ for all k = 1,…,K . The function $\rho_{\text{MUSIC}}\left(\theta \right)$ is usually referred to as a *pseudo* spectral density function since it does not strictly satisfy the generative model in Eq. (1).

#### 2.3.2 The case of TI-MRI

For $T_{1}$-weighted MRI with varying inversion-recovery (TI) times, the signal is modeled as

$$\begin{array}{ccc} s_{n}=\sum_{k=1}^{K}c_{k}\left(1-2e^{-TI_{n}/T_{1,k}}+e^{-TR/T_{1,k}}\right)+\epsilon_{n}, & & \end{array}$$ (24)

where repetition time (TR) is fixed, and multiple TIs are applied to probe the $T_{1}$ values of different tissue compartments. Eq. (24) can be written in a similar form as Eq. (18) below:

$$\begin{array}{ccc} s_{n}=c-\sum_{k=1}^{K}2c_{k}e^{-\theta_{k}x_{n}}+\epsilon_{n}, & & \end{array}$$ (25)

where $x_{n}\;=\;TI_{n}$ and $\theta_{k}\;=\;1/T_{1,k}$ and $c\;=\;\sum_{k=1}^{K}c_{k}(1+e^{-TR/T_{1,k}})$ . Eq. (25) can be considered a special case of Eq. (18) with an additional single component being constant. However, the constant term dominates the signal subspace, as indicated in the following equation:

$$H(\text{s})=H(\epsilon )+\left[\begin{array}{cccc} 1 & e^{-\theta_{1}x_{1}} & \ldots & e^{-\theta_{K}x_{1}}\\ 1 & e^{-\theta_{1}x_{2}} & \cdots & e^{-\theta_{K}x_{2}}\\ \vdots & \vdots & \vdots & \vdots \\ 1 & e^{-\theta_{1}x_{N-K}} & \cdots & e^{-\theta_{K}x_{N-k}} \end{array}\right]$$

$$\times \left[\begin{array}{cccc} c & c & \cdots & c\\ -2c_{1} & -2c_{1}e^{-\theta_{1}\delta_{x}} & \cdots & -2c_{1}e^{-K\theta_{1}\delta_{x}}\\ -2c_{2} & -2c_{2}e^{-\theta_{2}\delta_{x}} & \cdots & -2c_{2}e^{-K\theta_{2}\delta_{x}}\\ \vdots & \vdots & \vdots & \vdots \\ -2c_{K} & -2c_{K}e^{-\theta_{K}\delta_{x}} & \cdots & -2c_{2}e^{-K\theta_{K}\delta_{x}} \end{array}\right].$$ (26)

The dominant constant term in the signal subspace can significantly reduce the sensitivity of the subspace methods to detect exponential signals. To mitigate this limitation, we introduce the following orthogonal project matrix:

$$\Pi =I-1{\left(1^{T}1\right)}^{-1}1^{T},$$

which projects signals to the null space of the constant vector 1. Then, a modified MUSIC spectral density function is defined as

$$\begin{array}{ccc} \rho_{\text{MUSIC}}\left(\theta \right)=\frac{\text{e}{\left(\theta \right)}^{T}\Pi \text{e}\left(\theta \right)}{\text{e}{\left(\theta \right)}^{T}\Pi U_{n}U_{n}^{T}\Pi \text{e}\left(\theta \right)}, & & \end{array}$$ (27)

where both the noise subspace matrix and the testing vector are projected to the space of Π to remove the impact of the constant term on spectral analysis.

#### 2.3.3 Multidimensional MUSIC

The MUSIC method has been generalized to estimate multidimensional spectral analysis (Hua, 1993; Li et al., 1998; Liao, 2015). For simplicity, the method is introduced based on two-dimensional exponential variables $\theta_{k}=[\theta_{k,1};\theta_{k,2}]$ , with measurements on a two-dimensional regular grid $\text{x}\;=\;[x_{1,m};x_{2,n}]$ , for m = 1,...,M , and n = 1,...,N . Then, the measurements are represented as

$$\begin{array}{ccc} s_{mn}=\sum_{k=1}^{K}c_{k}e^{-\left[\theta_{k,1};\theta_{k,2}]\cdot [x_{1,m};x_{2,n}\right]}+\epsilon_{mn}, & & \end{array}$$ (28)

for m = 1,...,M and n = 1,...,N . For a given m, let

$${\text{s}}_{m,:}\;=\;[s_{m1};s_{m2};...;s_{mn}],$$

with $H(s_{m:})$ denoting the corresponding Hankel matrix. Next, the following 2-fold Hankel matrix is introduced

$$\begin{array}{c} H\left(\text{s}\right)=\left[\begin{array}{cccc} H({\text{s}}_{1,:}) & H({\text{s}}_{2,:}) & ... & H({\text{s}}_{K+1,:})\\ H({\text{s}}_{2,:}) & H({\text{s}}_{3,:}) & ... & H({\text{s}}_{K+2,:})\\ ⋰ & ⋰ & ⋰ & \vdots \\ H({\text{s}}_{M-K,:}) & H({\text{s}}_{M-K+1:}) & ... & H({\text{s}}_{M:}) \end{array}\right]. \end{array}$$ (29)

Accordingly, we define the following 2-fold signal vector on the measurement grid

$$\begin{array}{ccc} e\left(\theta \right)=\left[\begin{array}{c} e^{-\theta_{1}x_{1,1}}\\ e^{-\theta_{1}x_{1,2}}\\ \ldots \\ e^{-\theta_{1}x_{1,M-K}} \end{array}\right]⊗\left[\begin{array}{c} e^{-\theta_{2}x_{2,1}}\\ e^{-\theta_{2}x_{2,2}}\\ \ldots \\ e^{-\theta_{2}x_{2,N-K}} \end{array}\right], & & \end{array}$$

where ⊗ denotes the Kronecker product. Thus, by construction, $e(\theta_{k})$ belongs to the column subspace of the H(s) in the noiseless case.

Let $U_{n}$ denote the noise subspace matrix based on the singular value decomposition in the presence of measurement noise. The generalized MUSIC spectral density on the two-dimensional space is defined as

$$\begin{array}{ccc} \rho_{\text{MUSIC}}(\theta )=\frac{e{\left(\theta \right)}^{T}e(\theta )}{e{\left(\theta \right)}^{T}U_{n}U_{n}^{T}e(\theta )}. & & \end{array}$$ (30)

Similar methods can be used to generalize to d-dimensional spaces by using d-folder Hankle matrices. If the signal contains measurements with varying TI values, the method proposed in Eq. (27) can be used to improve spectral resolution with a suitable orthogonal projection matrix.

## 3 Experiments

### 3.1 Simulation data

The proposed algorithms are evaluated using two simulation datasets. The first dataset consists of a diffusion-weighted signal with multiple TEs based on the following two-compartment model:

$$s(b_{i},TE_{j})=\;\;\sum_{n=1}^{N}c_{n}e^{-b_{i}D_{n}-TE_{j}R_{2,n}},$$ (31)

where N=2 , $D_{1}/D_{2}=0.8/0.3\;\mu m^{2}/ms,\;R_{2,1}/R_{2,2}=18/12\;1/\text{s}$ (i.e., $T_{2,1}\approx 0.055\;\text{s}$ , $T_{2,2}\approx 0.083\;\text{s}$ ) and the two compartments have an equal volume fraction. The signals are sampled on a regular grid with $b=0,\;1,\;2,\;3,\;4,\;5\;ms/\mu m^{2}$ and TE=0.071, 0.101, 0.131, 0.161, 0.191 s . White Gaussian noise is added to the signals with different signal-to-noise ratios (SNR), that is, the ratio between the mean signal and the standard deviation of the noise, to investigate the sensitivity of the estimation methods with measurement noise.

The algorithms are further examined with variable parameters using signals generated based on the following model

$$\begin{array}{ccc} s(b_{i},TE_{j})=e^{-b_{i}D_{\text{fix}}-TE_{j}R_{2,\text{fix}}}+e^{-b_{i}D_{\text{mov}}-TE_{i}R_{2,\text{mov}}}, & & \end{array}$$ (32)

where the parameters for the first component are fixed with $D_{\text{fix}}=0.8\;\mu {\text{m}}^{2}/\text{ms}$ and $R_{2,\text{fix}}=18\;1/\text{s}$ . The parameters for the second component vary in the intervals $D_{\text{mov}}\in \left[0.1,\;1.6\right]\;\mu {\text{m}}^{2}\;\text{ms}$ and $R_{2,\text{mov}}\in \left[4,\;34\right]\;1/\text{s}$ , respectively, with 31 samples for each variable. Gaussian noise with SNR = 300 is added to the simulated signals for each set of parameters. This experiment evaluates the performance of different methods for separating the two components.

The proposed methods are applied to a three-dimensional dataset generated by the following model:

$$\begin{array}{ccc} s(b_{i},TE_{j},TI_{k})=\;\;\sum_{n=1}^{N}c_{n}(1-2e^{-TI_{k}R_{1,n}})e^{-b_{i}D_{n}-TE_{i}R_{2,n}}, & & \end{array}$$

where N=3 , $D_{1}/D_{2}/D_{3}=0.7/0.3/0.2\;\mu m^{2}/ms$ , $R_{2,1}/$ $R_{2,2}/R_{2,3}=18/13/30\;1/\text{s}$ , $R_{1,1}/R_{1,2},\;R_{1,3}=0.7/1.1/1.5\;1/\text{s}$ . The range of $R_{1}$ and $R_{2}$ values is selected based on the range of $T_{1}$ relaxation time of different brain tissue (Bojorquez et al., 2017; Stanisz et al., 2005; Wansapura et al., 1999). The signals are sampled on a regular grid with $b=0,\;0.75,\;1.4,\;2.1,\;2.8,\;3.5\;\text{ms}/\mu {\text{m}}^{2}$ and TE=0.07, 0.10,  0.13, 0.16, 0.19​​s and TI=0.3, 0.5, 0.7, 0.9, 1.1, 1.3​​s , respectively, and all three components have the same volume fraction. White Gaussian noise is added to the signal to investigate the sensitivity of the estimation methods to noise. This dataset provides insights to examine the benefit of using high-dimensional measures to resolve signals from different compartments. No variable parameters are investigated due to the large parameter space.

### 3.2 In vivo data

The proposed methods are applied to two *in vivo* datasets. The first dataset is from (Ning et al., 2020) which includes dMRI with multiple TEs acquired on a Siemens MAGNETOM Prisma 3T scanner with the following parameters: $b=0,\;0.75,\;1.4,\;2.1,\;2.8,\;3.5\;\text{ms}/\mu {\text{m}}^{2}$ with 30 non-collinear gradient directions for each non-zero b value, TE=0.071, 0.101, 0.131, 0.161, 0.191 s (same as simulation data), TR=5.9 s , SMS=2, iPAT=2, 2.5 mm isotropic voxels, and the matrix size is 96×96×54 . The acquired data were processed to correct for motion and distortion using FSL Topup/Eddy (Smith et al., 2004). The dataset includes a $T_{1}$-weighted MPRAGE. FreeSurfer (Fischl, 2012) is applied to obtain a tissue label map registered with the dMRI data using a rigid transform. The proposed methods are applied to estimate the $R_{2}-D$ distribution in various brain regions. The main goal of the analysis is to compare the different estimation methods and the microstructure across multiple brain regions.

The second *in vivo* example is based on the Multidimensional Diffusion MRI (MUDI) Challenge data (Pizzolato et al., 2020) from a 3T Philips Achieva Scanner acquired using the sequence developed in Hutter et al. (2018) with the following measurement parameters: b = 0, 0.5, 1, 2, 3 $\text{ms}/\mu {\text{m}}^{2}$ with 106 directions, 28 inversion times (IT) between 0.02 s and 7.322 s, TE = 0.08, 0.105, 0.130 s, TR = 7.5 s, 2.5 mm isotropic voxels, SMS = 2, SENSE = 1.9, matrix size 92×83​​​×55 , and a total of 1344 volumes. The acquired data are denoised and corrected for distortion as described in Hutter et al. (2018). The direction-averaged magnitude data are represented as

$$s\left(TI,TE,b\right)=\sum_{k=1}^{K}\;\left|(1-2e^{-TI\cdot R_{1,k}})e^{-b\cdot D_{k}-TE\cdot R_{2,k}^{*}}\right|,$$ (33)

where $R_{2}^{*}$ denotes the inverse of the $T_{2}^{*}$ relaxation time. The dependence of TR is ignored since TR is much longer than typical $T_{1}$ values. The MaxEnt and $LS_{+}$ methods are applied to estimate the joint distribution of $R_{1},R_{2}^{*}$, and D. The MUSIC approach is not applied in this dataset because of the limited number and nonuniform samples for b-values and TEs. More specifically, the MUSIC approach requires uniform samples for the construction of Hankel matrices. The size of the Hankel matrix in each dimension should be larger than the expected number of signals. In these data, the nonuniform b-values and the limited samples of TEs make it infeasible to construct the 3-fold Hankel matrices as in the simulation data.

### 3.3 Estimation methods

The proposed methods are compared with the solution of the following nonnegative least squares (${\text{LS}}_{+}$) method

$$\begin{array}{ccc} \underset{x\geq 0}{\text{min}}\frac{1}{2}∥A\text{x}-\text{s}{∥}_{2}^{2}+\;\frac{\sigma }{2}∥\text{x}{∥}_{2}^{2}, & & \end{array}$$ (34)

where each column of A represents the signal related to a specific tissue parameter. The estimated representation vector x provides a non-parametric characterization of the tissue parameters. To illustrate the dependence of the estimation results on the number of atoms in the basis, two ${\text{LS}}_{+}$ methods are implemented in the analysis. The first method consists of $10^{6}$ basis functions with 1000 samples for $R_{2}\in [0.3,\;3]\;1/\text{s}$ and 1000 samples for $D\in [0.001,\;3]\;\text{ms}/\mu {\text{m}}^{2}$ for the estimation of $T_{2}$-Diffusion distribution. The MaxEnt and the MUSIC methods are evaluated on the same set of grid points. The second method, named $LR-LS_{+}$, uses a basis matrix with $4\times 10^{4}$ samples, with 200 samples in $R_{2}$, and 200 samples in D on the same interval as above. The difference between $LS_{+}$ with a high-resolution grid and $LR-LS_{+}$ reveals the dependence of the basis-representation method on the resolution of basis functions.

For the estimation of the 3D $R_{1}$-$R_{2}$-D distributions in the simulation data, all three methods are evaluated on $8\times 10^{6}$ grid points with 200 samples in $R_{2}$, 200 samples in D as above, and 200 samples in $R_{1}\in [0.3,\;3]\;1/\text{s}$ . For 3D *in vivo* data, the $R_{1}-R_{2}^{*}-D$ distributions are estimated based on 100 samples in each dimension for $D\in [0.001,\;3]\;\text{ms}/\mu {\text{m}}^{2}$, $R_{2}^{*}\in [0.5,\;100]\;1/\text{s}$ , $R_{1}\in [0.33]\;1/\text{s}$ . The 3D results provide information to evaluate the importance of using high-dimensional RDD for microstructure analysis.

### 3.4 Evaluation metrics

The following metrics are used to examine the accuracy of the estimated density functions.

*Number of Peaks (NoP):* The number of dominant peaks of the estimated density functions is considered an estimate of the number of tissue compartments. The MATLAB-based *peaks2* function (Tikuišis, 2024) is applied to estimate the number of peaks in two-dimensional distributions. To mitigate the noise impact, a peak with a magnitude of at least 10% of the maximum value of the density function is considered a legitimate peak. For the second simulation dataset, the estimated 3D density functions are projected to 2D marginals of the $R_{2}-D$ , $R_{1}-D$ , and $R_{1}-R_{2}$ spaces, and the average number of peaks in the three marginals is considered as the estimated number of components.

*Peak Distance:* The Peak Distance is computed to investigate the bias of the peaks of the estimated density functions based on the following equation

$$\begin{array}{c} \text{Peak Distance}=\frac{1}{N}\;\sum_{i=1}^{N}\underset{j}{\text{min}}∥\theta_{\text{true},i}-\theta_{\text{est},j}{∥}_{1}, \end{array}$$ (35)

where $\theta_{\text{true},i}$ represents the true parameter vector of the i-th components and $\theta_{\text{peak},j}$ of the j-th peak. To reduce the bias of the error metric related to the difference in units, the parameters are all linearly scaled to the [0, 1] unit interval for comparison.

*Wasserstein Distance:* The Wasserstein distance, that is, the optimal mass transport distance, is a standard metric to evaluate the difference between density functions (Jiang et al., 2012; Kolouri et al., 2017). Let $\rho_{\text{true}}\left(\theta \right)$ and $\rho_{\text{est}}\left(\theta \right)$ denote the true and estimated density functions that are normalized with the same integral. Let $c(\theta_{i},\theta_{j})=\;∥\theta_{i}-\theta_{j}{∥}_{2}$ denote the cost for transporting one unit of mass from $\theta_{i}$ to $\theta_{j}$. Then, the Wasserstein-1 metric between the two density functions is defined as

$$\begin{array}{c} W_{1}\left(\rho_{\text{true}},\rho_{\text{est}}\right)=\underset{\gamma \geq 0}{\text{min}}\int \int \gamma \left(\theta_{1},\theta_{2}\right)c\left(\theta_{1},\theta_{2}\right)d\theta_{1}d\theta_{2}\\ \text{s}.t.\int \gamma \left(\theta_{1},\theta_{2}\right)d\theta_{2}=\rho_{\text{true}}\left(\theta_{1}\right),\int \gamma \left(\theta_{1},\theta_{2}\right)d\theta_{1}=\rho_{\text{est}}\left(\theta_{2}\right). \end{array}$$ (36)

Compared to the Peak Distance metric, the Wasserstein distance between the true and estimated density functions is sensitive to the bias in peak locations and the spread of the mass around the peaks. Moreover, it does not depend on any peak detection algorithm, providing an alternative and potentially more robust metric to evaluate the bias in the estimated density functions.

## 4 Results

### 4.1 2D simulation results

**Fig. 1.**
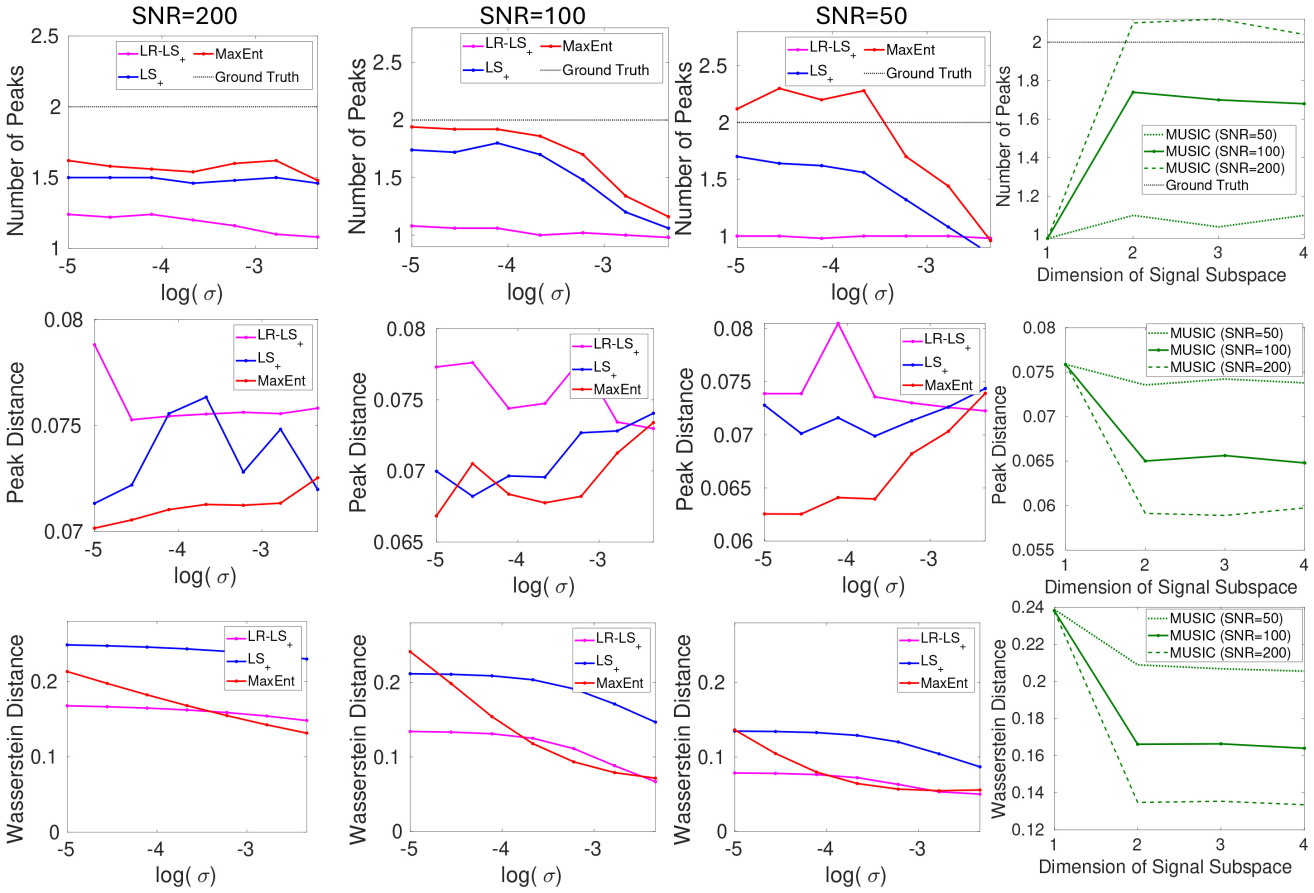
The performance MaxEnt, $LS_{+}$, $LR-LS_{+}$ and MUSIC methods for the estimation of two-component $R_{2}-D$ distributions using simulation data.

Figure 1 illustrates the performance of the estimation methods related to different levels of SNR, shown in various types of plots with different free parameters in the algorithm on the x-axis. The first row shows the Number of Peaks (NoP) for the four methods averaged over 100 independent trials for different log(σ) values. For reference, the heuristic values for log(σ) are equal to -3.26, -4.6 and -6.03 for SNR = 50, 100, 150, respectively. The MaxEnt and (high-resolution) $LS_{+}$ have very similar performance with accurate estimation results in high SNR stimulation with relatively weak regularization parameters and underestimated NoP with a lower SNR. The $LR-LS_{+}$ has underestimated NoP even with a high SNR. The MUSIC approach is sensitive to measurement noise, with accurate estimation with SNR = 200. The performance of MUSIC is stable as long as the dimension of the signal subspace is larger than the actual number of signals.

The second row of Figure 1 shows the Peak Distance metrics. For MaxEnt and $LS_{+}$, the metrics increase with lower SNR and stronger regularization parameters. The MaxEnt has a lower error than the $LS_{+}$ approach. The $LR-LS_{+}$ method has a more biased estimation than the $LS_{+}$ approach. The MUSIC method has the most accurate estimation at high SNR. The last row of Figure 1 shows the Wasserstein Distance between the true and estimated distributions. For MaxEnt and $LS_{+}$, the distance increases with stronger regularization parameters, indicating that the peaks are merged but with the mass spread around the true parameters, while the number of peaks decreases. The MUSIC method has a higher distance error since it does not estimate the true distributions.

**Fig. 2.**
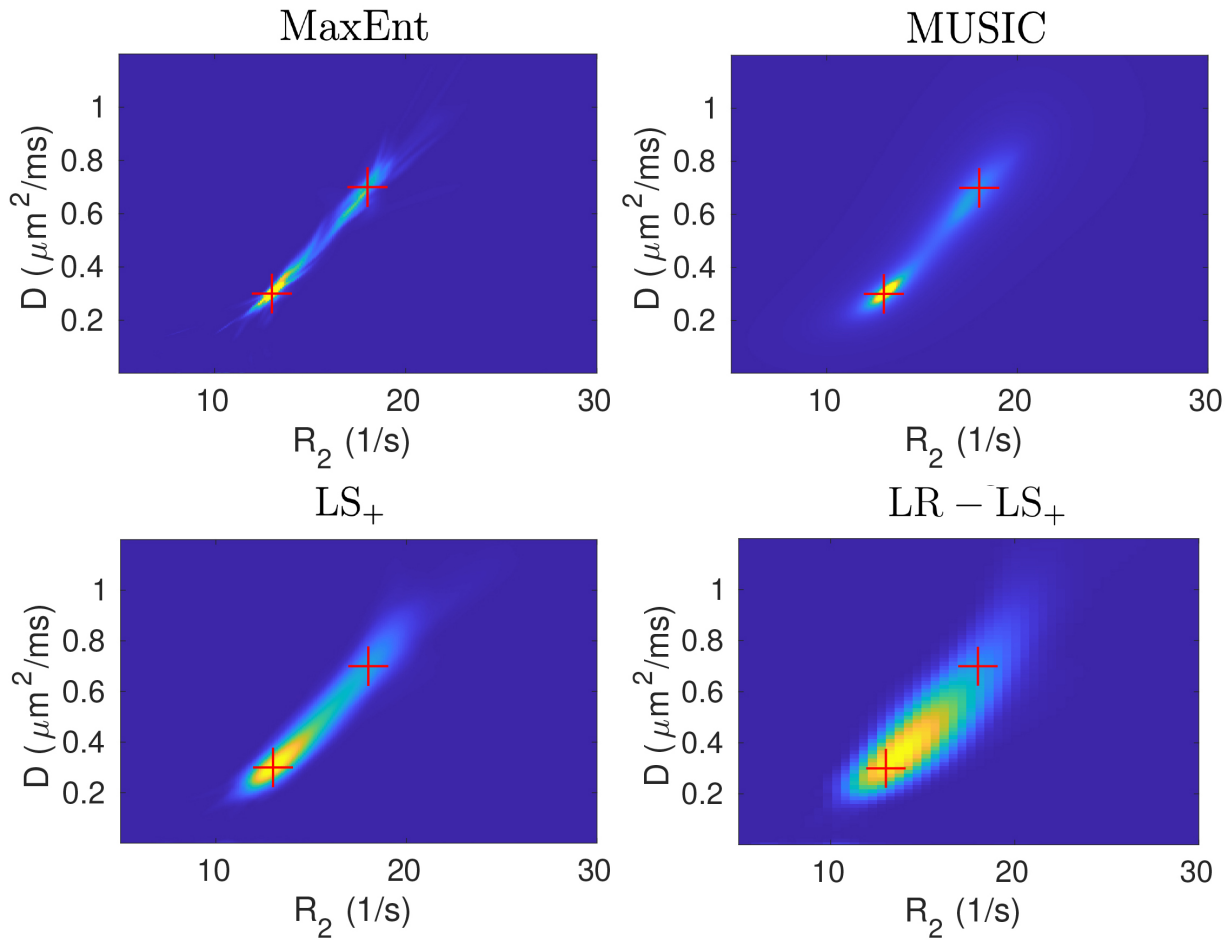
Illustration of the mean $R_{2}-D$ density functions for the MaxEnt, $LS_{+}$, $LR-LS_{+}$ and the MUSIC method for data with SNR = 200.

Figure 2 illustrates the mean density function for the four methods based on data with SNR = 200, with the red crosses showing the true parameters. Both MaxEnt and MUSIC show two modes. The $LS_{+}$ methods show one dominant peak because of the average effect between multiple density functions with variable peak locations and more spread distributions. The $LR-LS_{+}$ failed to detect the second peak, indicating the importance of using high-resolution basis matrices. On average, the MaxEnt and the $LS_{+}$ method take 11.88 and 114.7 s computation time with MATLAB R2022 on a Linux workstation (Intel Xeon Gold 5318Y), but the MUSIC method only takes 0.1 s.

**Fig. 3.**
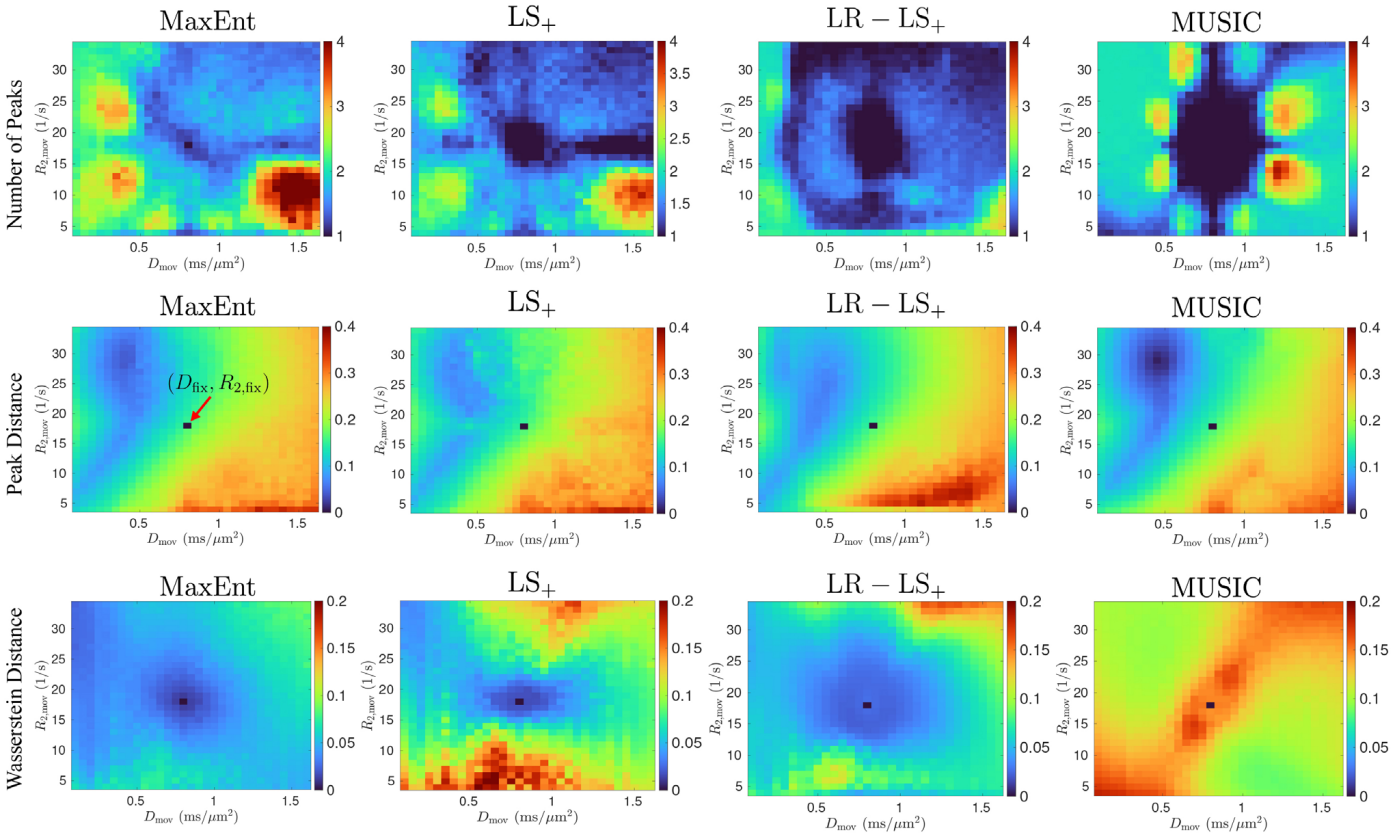
Illustration of the error metrics for $R_{2}-D$ density functions for two-component signals with moving parameters generated using Eq. (32) with SNR = 300.

Figure 3 illustrates the error metrics based on signals generated using a two-component model (32) with moving parameters. The first row shows the NoP maps for the four methods, where the two axes correspond to $D_{\text{mov}}$ and $R_{2,\text{mov}}$ of the moving parameters. The metrics at each point of the image reflect the average values from 100 independent trials, which mitigates the impact of noise samples. The MaxEnt method shows a more accurate number of peaks than other methods, especially in the regions around the fixed parameters, indicating better spectral resolution. The image shows nonuniform metrics that depend on the relative locations of the parameters. The $LS_{+}$ method only detects one peak in regions around the fixed parameters, indicating the reduced spectral resolution compared to the MaxEnt method. The $LR-LS_{+}$ method shows significantly underestimated NoP values, highlighting the need for a high-resolution basis for $R_{2}-D$ analysis. The MUSIC method displays different spatial patterns than the MaxEnt and $LS_{+}$ methods, with a larger area with underestimated NoP values but fewer areas with overestimated NoP values.

The second row of Figure 3 illustrates the Peak Distance error metrics, where the red arrow highlights the fixed parameters. All four methods have similar spatial distributions of the error metrics. The MUSIC method shows a lower value, indicating more accurate estimation if the two peaks are well separated.

The last row of Figure 3 illustrates the Wasserstein Distance between the estimated and true distributions. The MaxEnt method shows lower values than the other three methods, even in regions with overestimated numbers of peaks. The MUSIC method has higher errors than other methods, which is expected since the MUSIC method provides a pseudo distribution that does not satisfy measurements. The MaxEnt approach provides more robust and accurate $R_{2}-D$ distributions than other methods.

### 4.2 3D simulation results

**Fig. 4.**
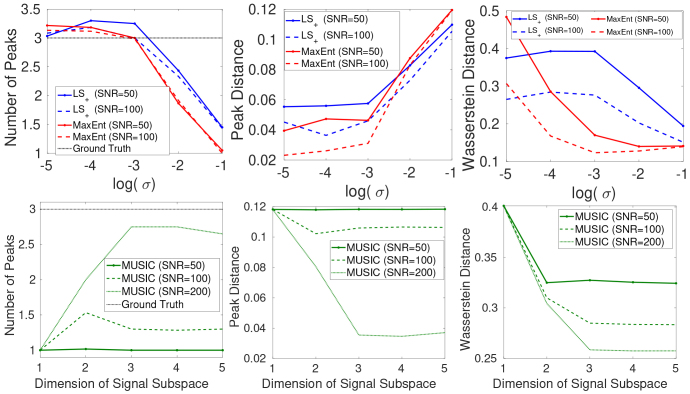
The performance MaxEnt, $LS_{+}$, $LR-LS_{+}$ and MUSIC methods for the estimation of three-component $R_{1}-R_{2}-D$ distributions using simulation data.

The first row of Figure 4 shows the performance metrics of the MaxEnt and $LS_{+}$ for estimating three-dimensional density functions with three components. A key result is that the MaxEnt and $LS_{+}$ perform significantly better than the two-dimensional case in Figure 1 at SNR = 50. Specifically, MaxEnt and $LS_{+}$ have accurate NoP estimations for a wide range of regularization parameters. The MaxEnt method still has lower Peak Distance and Wasserstein Distance metrics than the $LS_{+}$ method, indicating a more accurate estimation of the density functions.

The second row of Figure 4 shows the results for the MUSIC method. Similar to results in Figure 1, the performance strongly depends on the SNR. The MUSIC method accurately estimates the NoP only at very high SNR. The performance degrades significantly with lower SNR values.

**Fig. 5.**
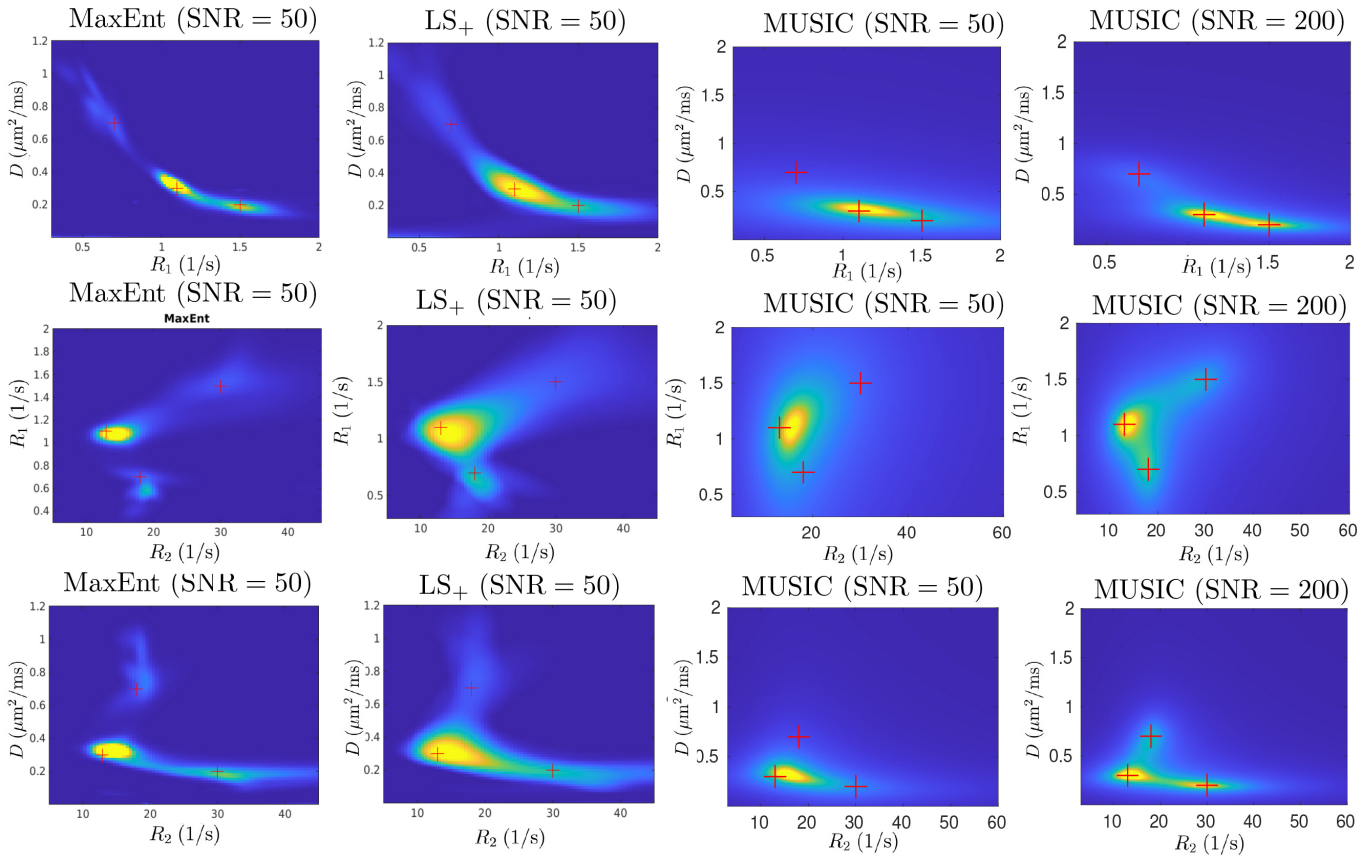
Illustration of the two-dimensional marginals (first row: $R_{1}-D$ distribution, second row: $R_{2}-R_{1}$, third row: $R_{2}-D$ ) of the estimated $R_{1}-R_{2}-D$ density functions for the MaxEnt, $LS_{+}$ and the MUSIC method.

Figure 5 illustrates the two-dimensional marginal of the estimated $R_{1}-R_{2}-D$ three-dimensional distributions. The first two columns show the results for the MaxEnt and $LS_{+}$ methods, respectively, where the red crosses indicate the location of the true parameters. Both methods show three components in all 2D marginals. The MUSIC approach only shows one component at SNR = 50. The three components are revealed with a higher SNR = 200, as shown in the last column. Due to increased data size, the MaxEnt and the $LS_{+}$ method take much longer computation times, with 619 and 1019 s, respectively. The increased computation time for the MaxEnt method is mainly related to the integral in the computation for the gradients and Hessian. The $LS_{+}$ method is more demanding on the memory related to the inversion of a large matrix, which limits the highest resolution in this example. The MUSIC method is the most time-efficient approach and only takes 1.4 s per trial.

In summary, the results in this example show that the MaxEnt and $LS_{+}$ methods perform much better in separating multiple components in three-dimensional space than in two-dimensional spaces, especially in situations with low SNR. The MUSIC method accurately estimates data with high SNR if a sufficiently large number of signal subspaces is selected.

### 4.3 2D in vivo data results

**Fig. 6.**
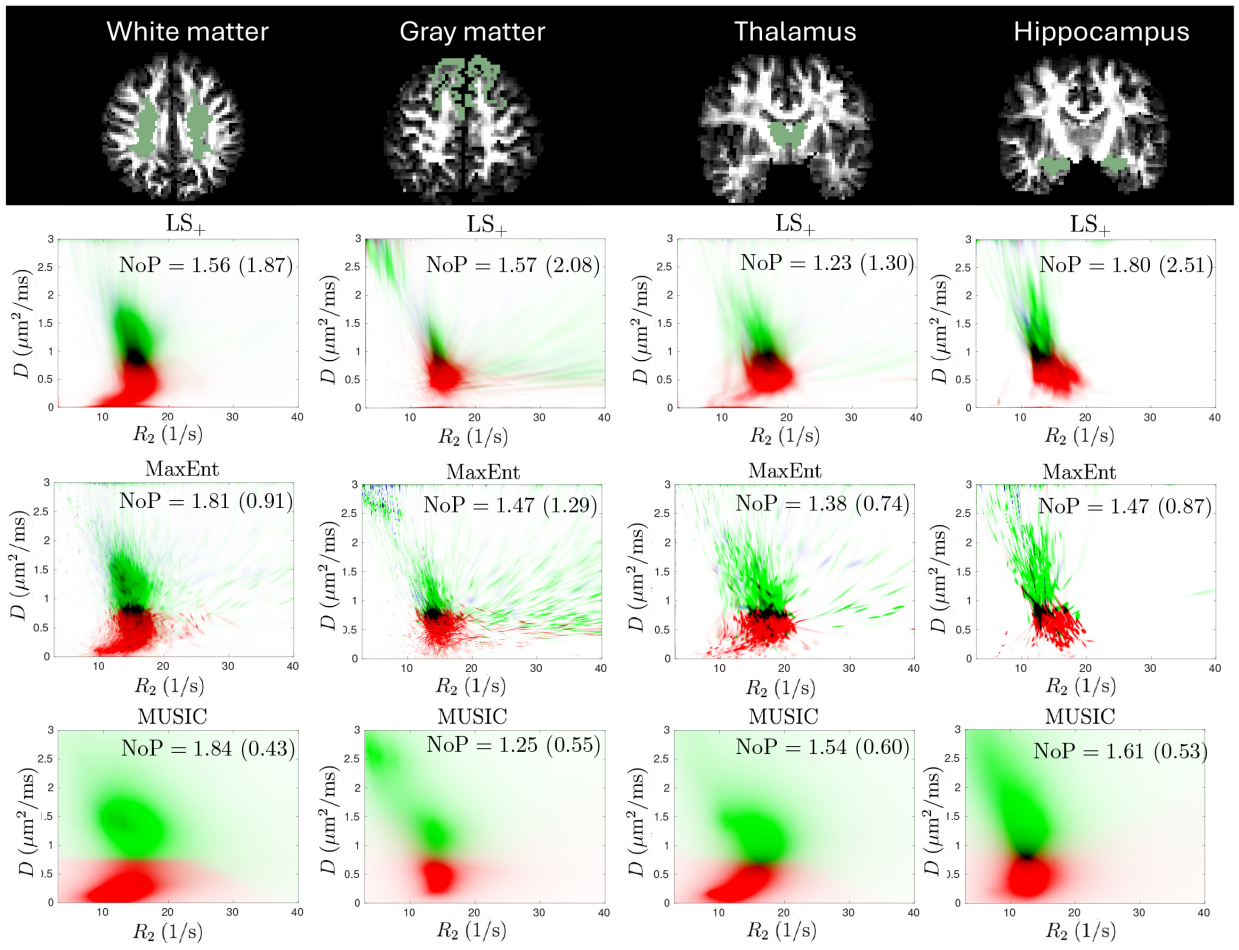
Comparison of the $LS_{+}$ method (second row) and the proposed MaxEnt (third row) and MUSIC (fourth row) methods using *in vivo* MRI data acquired in a two-dimensional space of $R_{2}$ and D for different brain regions. Different components in each voxel are coded in different colors.

Figure 6 illustrates the estimated $R_{2}-D$ distributions averaged over multiple voxels in several brain regions. The first row illustrates the FreeSurfer-based labels for these regions overlaid on the $T_{1w}$ image. The second to the fourth rows show the estimated $R_{2}-D$ distributions from the $LS_{+}$, MaxEnt, and MUSIC methods, respectively, averaged over all voxels in the selected brain regions. If the RDD distribution has multiple peaks detected by the *peaks2* toolbox (Tikuišis, 2024), then the modes are classified into different components based on the underlying diffusivity. For example, the component with the lowest diffusivity is encoded by red and the green color encodes components with higher diffusivity. The black color highlights the overlaps between red and green colors over multiple voxels in the selected brain region.

All three methods reveal two tissue components in the mean density maps. The gray matter region also includes the third component with a high diffusivity around 3 $\mu m^{2}/ms$ and low $R_{2}$ (long $T_{2}$), which is potentially related to the cerebrospinal fluid caused by the partial volume effect. Overall, the MaxEnt approach shows density maps and estimated mean NoP values similar to the $LS_{+}$ method but with much lower standard deviation values. The MUSIC method has the lowest variability in NOP measures and smoother density maps than the other methods. To examine the reliability of the results, the SNR is estimated using the denoising method in Veraart et al. (2016), which shows the estimated average SNR in the four regions in the first row of Figure 6 are 162, 280, 123, 148, respectively. Although the true value of SNR is unknown, the large estimated value indicates reliable estimation results. However, the estimated density maps show significant variability within each brain region, especially in the gray matter and thalamus, indicating heterogeneous microstructure across the voxels.

**Fig. 7.**
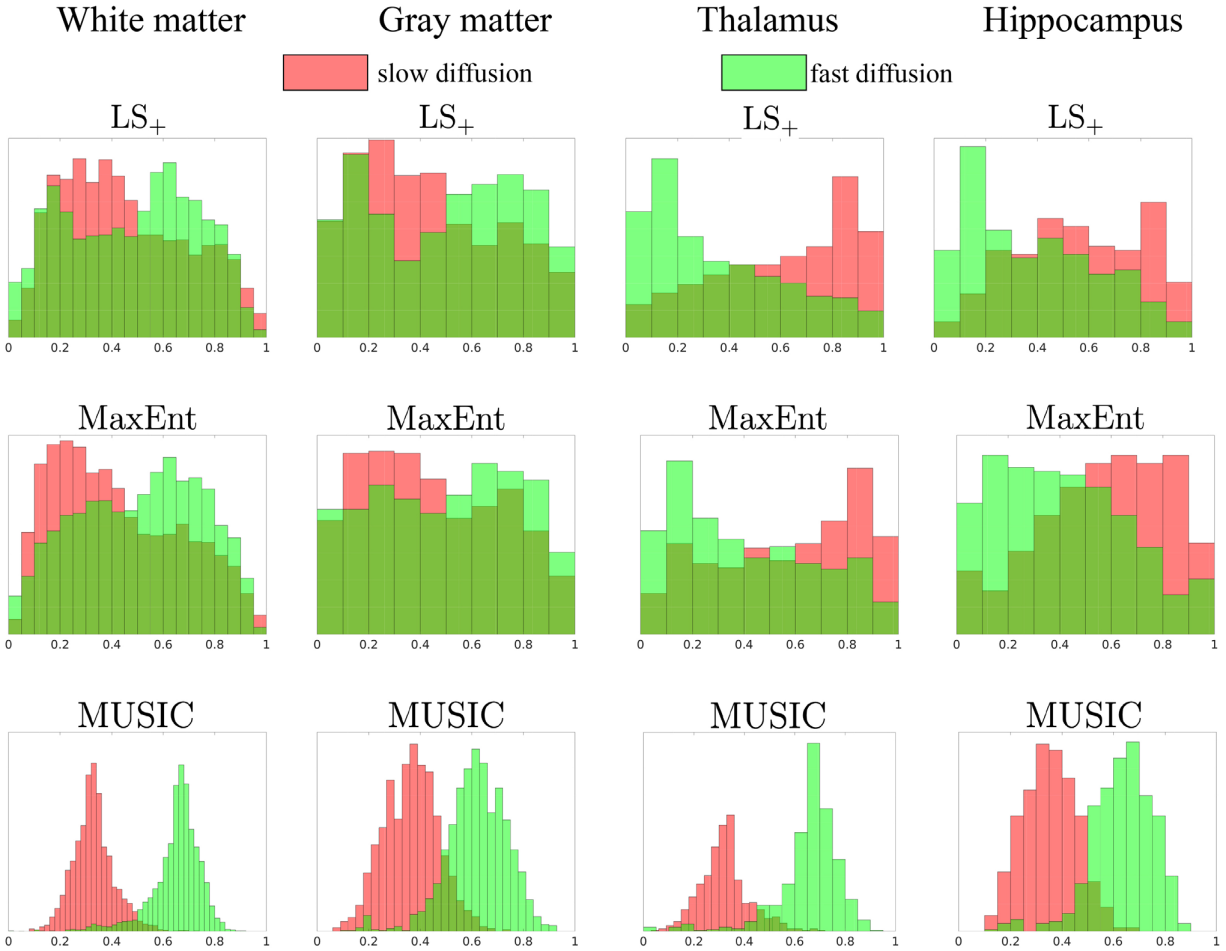
Histogram of the estimated volume fraction for the components with slow (red) and fast (green) diffusivity.

Figure 7 shows the histogram of the estimated volume fractions for the components highlighted in red (slow diffusivity) and green colors (fast diffusivity) in Figure 6. The volume fraction is computed as the ratio of the integral of the $R_{2}-D$ distribution in the different subsets corresponding to the red and green components, divided by the total integral, which takes values between 0 and 1. The first row shows the histogram corresponding to the $LS_{+}$ method. The cortical gray matter and white matter regions, the slow-diffusion components, have a lower volume fraction than the fast-diffusion components. The peak distributions for the slow and fast distributions in white and gray matter are around (0.3, 0.65) and (0.3, 0.7), respectively. The thalamus and hippocampus regions show a reverse trend with higher volume fractions for the low-diffusion components and lower values for the fast-diffusion components. The peak distributions for the slow and fast distributions in both regions are around (0.8, 0.2).

The second row shows the histogram for the MaxEnt method. The distributions are similar to the results from the $LS_{+}$ method shown in the first row. In the white matter, the volume fraction for slow-diffusion components (red) has a slightly lower peak value around 0.2 compared to the $LS_{+}$ method.

The last row shows the volume fractions for the MUSIC method. Since the MUSIC method provides a pseudo distribution that provides an accurate estimation of the number of peaks and peak locations in some cases, but with a biased mass distribution as shown in Figure 3, the volume fractions are very different from the results of the other two methods, as expected.

It is noted that the two components are only separated based on the relative values of diffusivity, which may include various components in each category. Thus, each category is expected to include heterogeneous distributions of volume fractions.

### 4.4 3D in vivo data results

**Fig. 8.**
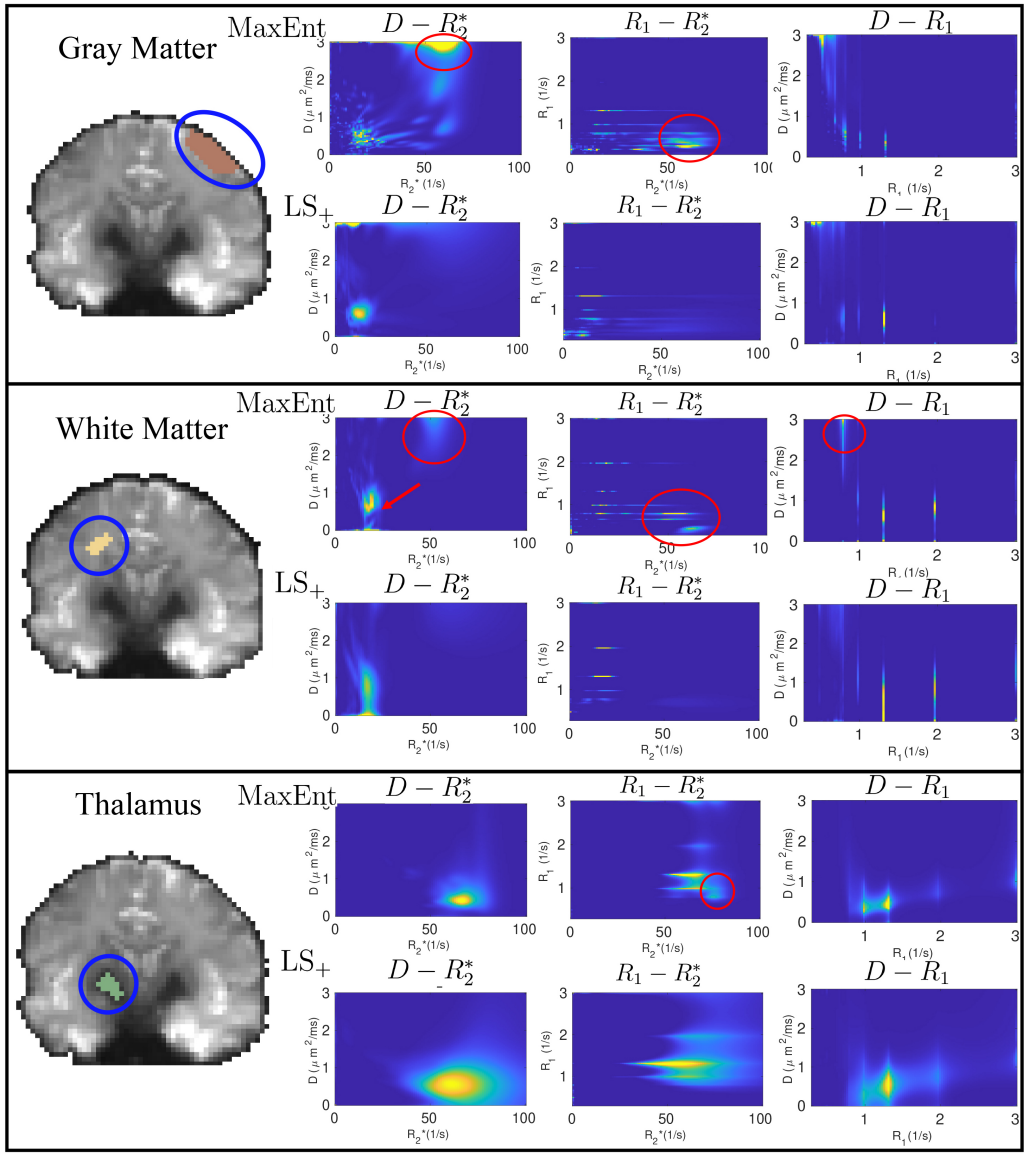
Comparison of the three dimensional $R_{1}-R_{2}^{\text{*}}-D$ distributions estimated using the MaxEnt and $LS_{+}$ methods for three brain regions. The red circles and arrows highlight the main differences between the two methods.

Figure 8 illustrates the average $R_{1}-R_{2}^{*}-D$ distributions in three manually selected brain regions in the cortical gray matter, white matter, and the thalamus, respectively. The top panel shows the results for the gray matter region, highlighted by the blue circle, where the upper and lower rows show the results for the MaxEnt and the $LS_{+}$ methods, respectively. The three columns show the $D-R_{2}^{*}$, $R_{1}-R_{2}^{*}$ and $D-R_{1}$ marginal distributions. Both methods show the same components with low diffusivity with *D* = 0.7 $ms/\mu m^{2}$, $R_{2}^{*}\in [10,\;25]\;1/\text{s}$ and distributed in three clusters in the $R_{1}$ space, due to improved resolution in the $R_{1}$ space related to more TI samples, which are potentially related to different restricted/hindered water components. The CSF component is also reviewed in both methods, which is characterized by high D and low $R_{2}^{*},R_{1}$ values. But the MaxEnt method reveals an additional component, highlighted by the red circles in the first row, that is not shown in the $LS_{+}$. This component is characterized by a high diffusivity, high $R_{2}^{*}$ mainly between 40 1/s and 60 1/s, and $R_{1}$ values fall between those of CSF and tissue components. These measures are consistent with the $R_{2}^{*}$ values of deoxygenated blood reported in Zhao et al. (2007) and the $T_{1}$ value of blood around 1.7 s in X. Zhang et al. (2013).

The second panel shows the estimated distributions in a white matter region. The MaxEnt and $LS_{+}$ methods show similar components with restricted/hindered diffusivity and low $R_{2}^{*}$ with multiple $R_{1}$ values. The red arrow in the $D-R_{2}^{*}$ image shows that the MaxEnt method shows higher resolution with two separated components that are not clearly separated in the $LS_{+}$ results. Moreover, red circles show one component revealed by the MaxEnt method that is not shown in the $LS_{+}$ results. This component has similar $R_{1},R_{2}^{*}$, and D measures as in the components shown by circles in the first row, which are potentially related to blood components in the perivascular spaces. This component is associated with high $R_{2}^{*}$ and D values, which significantly reduce the signal contributions. But its different $R_{1}$ value makes it possible to separate the signal from other components with varying TI values. Further investigations are needed to examine the reliability and the signal source of this component.

The last panel shows the distributions in a region of the thalamus. Both methods show similar results where the restricted/hindered component has higher $R_{2,*}$ values than the gray and white matter. The MaxEnt method shows higher resolution, which reveals more details in the distribution as highlighted in the red circle. The SNR for the three regions based on the noise map estimated using the MP-PCA denoising method (Veraart et al., 2016) are 87, 94, and 18, respectively.

## 5 Discussion

The proposed MaxEnt and MUSIC methods have several theoretical and practical advantages compared to the basis-representation technique with $LS_{+}$ estimation methods. Specifically, the MaxEnt result shows more accurate peak locations and less biased density functions regarding the Wasserstein distance than the $LS_{+}$ approach in simulations. Moreover, the MaxEnt method has better computation efficiency than high-resolution $LS_{+}$ by solving a convex optimization problem in a low-dimensional dual space without using a large inversion matrix.

For *in vivo* data, the MaxEnt method shows a reduced variability in the NoP measures compared to the $LS_{+}$ approach. The results also show that high-dimensional correlation-spectroscopy with the MaxEnt method provides higher spectral resolution than low-dimensional methods to detect multiple tissue components, even with noisy data. On the other hand, the MUSIC approach is the most efficient algorithm since it does not require the solution of an optimization problem. Moreover, its performance is not sensitive to hyperparameters as long as the dimension of the signal subspace is sufficiently large. However, the performance of the MUSIC approach is sensitive to measurement noise. The MaxEnt and $LS_{+}$ better estimate high-dimensional density functions with noisy data than the MUSIC approach. The MUSIC method shows similar NoP measures for *in vivo* data since the direction-averaged dMRI data have sufficiently high SNRs. Furthermore, the 3D $R_{1}-R_{2}^{*}-D$ distributions have revealed more tissue components than 2D results. Although further validations are needed, high-dimensional RDD analysis provides a promising approach for tissue microstructure analysis.

We note several limitations of the proposed methods and results. First, the modified MaxEnt method in Eq. (7) assumes that the measurement noise is Gaussian, which is usually not satisfied in MRI and depends on the image reconstruction algorithm (Cárdenas-Blanco et al., 2008; Gudbjartsson & Patz, 1995). An extension of the method for other noise distributions needs to be explored. Second, the ground truth for *in vivo* data is unknown. Further validations are needed to investigate the biological basis of the RDD functions using histology data as in Benjamini et al. (2023). Third, the dependence of the estimation results on the measurement parameters is also needed in future work to guide the most time-efficient sampling schedule. Previous works in Kim et al. (2017), Liu et al. (2025), and Slator et al. (2021) have shown that applying a suitable spatial regularization or sparsity-promoting norms improves the linear inverse method for RDD estimation. Other methods have been proposed for RDD estimation using sparsely sampled data (Benjamini & Basser, 2016; Benjamini et al., 2023; de Almeida Martins & Topgaard, 2018; Manninen et al., 2024; Martin et al., 2021). Optimization of the MaxEnt method with sparsely sampled data will be examined in future work. Although the MUSIC approach requires samples on a regular grid, the performance of the MaxEnt method with sparsely sampled data with be examined in future work. Moreover, this work focuses on RDD estimation for scalar-valued diffusivity, which ignores direction-dependent diffusivity. The MaxEnt method is also feasible for the estimation of multidimensional diffusion tensor distributions as studied in Topgaard (2017, 2019). An improved sampling method, such as B-tensor encoding (Topgaard, 2019; Westin et al., 2016), and more efficient algorithms, such as the Monte Carlo inversion method (Benjamini & Basser, 2016; Benjamini et al., 2023; de Almeida Martins & Topgaard, 2018; Manninen et al., 2024; Martin et al., 2021), may be useful to further improve the accuracy and efficiency for computing the distributions in high-dimensional spaces.

In summary, the proposed MaxEnt and MUSIC algorithms are computationally efficient methods for correlation spectroscopy analysis with potentially better performance than basis-representation methods.

## Data Availability

The code for the proposed methods is available at https://github.com/LipengNing/ME-RDD.
